# β-Amyloid 1-42 Oligomers Impair Function of Human Embryonic Stem Cell-Derived Forebrain Cholinergic Neurons

**DOI:** 10.1371/journal.pone.0015600

**Published:** 2010-12-17

**Authors:** Linn Wicklund, Richardson N. Leão, Anne-Marie Strömberg, Malahat Mousavi, Outi Hovatta, Agneta Nordberg, Amelia Marutle

**Affiliations:** 1 Department of Neurobiology, Care Sciences and Society, Division of Alzheimer Neurobiology, Karolinska Institutet, Stockholm, Sweden; 2 Department of Neuroscience, Neuronal Oscillation Laboratory, Karolinska Institutet, Stockholm, Sweden; 3 Department of Clinical Science, Intervention and Technology, Division of Obstetrics and Gynecology, Karolinska Institutet, Karolinska University Hospital Huddinge, Stockholm, Sweden; 4 Department of Geriatric Medicine, Karolinska University Hospital Huddinge, Stockholm, Sweden; University of North Dakota, United States of America

## Abstract

Cognitive impairment in Alzheimer's disease (AD) patients is associated with a decline in the levels of growth factors, impairment of axonal transport and marked degeneration of basal forebrain cholinergic neurons (BFCNs). Neurogenesis persists in the adult human brain, and the stimulation of regenerative processes in the CNS is an attractive prospect for neuroreplacement therapy in neurodegenerative diseases such as AD. Currently, it is still not clear how the pathophysiological environment in the AD brain affects stem cell biology. Previous studies investigating the effects of the β-amyloid (Aβ) peptide on neurogenesis have been inconclusive, since both neurogenic and neurotoxic effects on progenitor cell populations have been reported. In this study, we treated pluripotent human embryonic stem (hES) cells with nerve growth factor (NGF) as well as with fibrillar and oligomeric Aβ_1-40_ and Aβ_1-42_ (nM-µM concentrations) and thereafter studied the differentiation *in vitro* during 28-35 days. The process applied real time quantitative PCR, immunocytochemistry as well as functional studies of intracellular calcium signaling. Treatment with NGF promoted the differentiation into functionally mature BFCNs. In comparison to untreated cells, oligomeric Aβ_1–40_ increased the number of functional neurons, whereas oligomeric Aβ_1–42_ suppressed the number of functional neurons. Interestingly, oligomeric Aβ exposure did not influence the number of hES cell-derived neurons compared with untreated cells, while in contrast fibrillar Aβ_1–40_ and Aβ_1–42_ induced gliogenesis. These findings indicate that Aβ_1–42_ oligomers may impair the function of stem cell-derived neurons. We propose that it may be possible for future AD therapies to promote the maturation of functional stem cell-derived neurons by altering the brain microenvironment with trophic support and by targeting different aggregation forms of Aβ.

## Introduction

Neurogenesis is thought to persist in the adult mammalian brain [1], but declines during ageing and is insufficient in preventing the neuronal loss that occurs in neurodegenerative disorders, such as Alzheimer's disease (AD). In AD, there is a marked reduction of basal forebrain cholinergic neurons (BFCNs), which correlates with the memory impairment and cognitive dysfunction observed in AD patients [2]. Nerve growth factor (NGF), a member of the neurotrophin family, promotes the survival of BFCNs by acting on their high affinity tyrosine kinase receptors (TrkAs) [3]. During the progression of the disease, NGF levels in the brain decrease as a result of dysmetabolism and impaired axonal transport [4], [5]. It has also been hypothesized that a diminished conversion of the precursor form of NGF (proNGF) to mature NGF, as well as augmented degradation of the mature form, could underlie the cholinergic atrophy observed in the AD brain [6].

The accumulation of β-amyloid (Aβ) plaques is a key feature in the brains of AD patients and implicated in the disruption of normal cellular processes leading to neurodegeneration [7]. During disease progression, Aβ peptides assemble into various aggregation forms, ranging from dimers and oligomers to fibrils in amyloid plaques. However, the magnitude of amyloid plaque deposition in the brain correlates poorly with cognitive decline, and emerging evidence suggests that Aβ oligomers may be the major culprits in this regard [8]. Functional studies have demonstrated that oligomeric Aβ species can impair long-term potentiation (LTP) and synaptic function in mature neurons [9].

Although neuronal loss is persistent in AD, an increased hippocampal neurogenesis has been reported in AD post mortem brain [10]. This exciting finding has reinforced the expectation that stimulating regenerative processes and cell survival in the brain may be clinically beneficial as a novel treatment approach for AD. Pluripotent human embryonic stem (hES) cells represent a rich source of expandable cells that can be used for generating various cell populations, including neurons. A concern regarding the therapeutic value of stem cells is identifying the conditions *in vivo* under which these cells differentiate into a specific lineage, and to develop reliable and reproducible protocols that would efficiently produce functionally mature neurons derived from stem cells.

In an earlier report, we showed that hES cells differentiate into neurons in feeder-free and serum-free conditions [11]. We have also recently established an optimized embryoid bodies based protocol that can generate neurons expressing functional cholinergic receptors following growth factor treatment [12]. Few studies to date have investigated the effects of Aβ on stem cell proliferation and differentiation and the existing data remains inconclusive [13], [14], [15], [16], [17]. Systematic studies investigating how fibrillar and oligomeric forms of Aβ influence the differentiation and functionality of human stem cells are therefore important.

In the present study, we treated hES cells with NGF to promote the differentiation into BFCNs. Furthermore, we examined the influence of fibrillar and oligomeric Aβ_1–40_ and Aβ_1–42_ on hES cell proliferation, cell survival signaling pathways and neuronal differentiation as well as the effects on cytosolic calcium [Ca^2+^]_i_.

## Materials and Methods

### Ethics statement

The lines HS293 and HS346 were previously derived from fresh poor quality embryos that had been donated for research in the Fertility Unit of the Karolinska University Hospital, Huddinge, Sweden. An informed consent form was signed by both partners after receiving an oral and written description of the study. The Ethics Board of the Karolinska Institutet approved the derivation and research use of these lines.

### Human embryonic stem cell culture

Two fully characterized hES cell lines, HS293 and HS346, were used in this study. Both lines have been derived from donated supernumerary blastocyst stage embryos with approval from the Ethics Board at the Karolinska Institutet, Sweden. Their derivation has been described in detail previously [18], [19], [20], [21], [22]. These lines have been accepted for use in the European Union (EU) projects by the European Commission, which accepted them for the integrated project ESTOOLS (www.estools.eu). They are also included in the EU Human Embryonic Stem Cell Registry (www.hESCreg.eu). The cell lines express pluripotency markers as described in the above-mentioned and other articles in our publications. Both lines express Oct 3/4, Nanog, SSEA3 and 4, Tra-1-81, Tra-1-60, and alkaline phosphatase. These hES lines have remained chromosomally stable up to high passage, >100, HS293 has a karyotype 46, XY and HS346 46, XX. When injected into immune deficient animals, they form teratomas, which contain components of all three germ layers. Figure S1 shows a panel of these characterization results (Figure S1). Both lines have also been gene expression profiled by Affymetrix human gene chip 133, microarray and by single nucleotide polymorphism array (Affymetrix 60).

HS293 and HS346 were grown on human foreskin fibroblasts, and colonies of cells were subsequently removed from the feeder layer and the culture was expanded at 37°C in a 5% CO_2_ humidified incubation chamber in serum-free DMEM/F12+ glutamax medium supplemented with B27 (1∶50) heparin (5 µg/ml), antibiotic-antimycotic mixture (1∶100) and EGF + bFGF (20 ng/ml each, Sigma, St. Louis, MO, USA). The cells were propagated as free-floating neurospheres and mechanically passaged every 2–3 weeks. The lines expressed neural lineage markers nestin, Sox1, and Pax6 when differentiated either as spheres or in adherent culture for seven days, and two weeks later vimentin, BLBP, GLAST, and GFAP as reported earlier [11]. For the *in vitro* differentiation experiments, neurospheres were plated in 6-well tissue culture plates following a stepwise protocol: culture in neuronal induction medium (NIM) consisting of DMEM/F12+ glutamax: Neurobasal medium, B27 (without vitamin A; 1∶50) and N2 supplement (1∶100) for 4 days. From day 4, the cells that adhered to the tissue wells were allowed to continue differentiation for another 18–35 days in neural proliferation medium (NPM) consisting of DMEM/F12+ glutamax: Neurobasal medium, N2 supplement (1∶200), B27 (1∶100) and bFGF (20 ng/ml). During differentiation, the media was replaced twice every week, and the cells were treated with NGF (50 ng/ml) or oligomeric (rPeptide, Bogart, GA, USA) and fibrillar (Sigma, St. Louis, MO, USA) Aβ_1–40_ and Aβ_1–42_ (100 nM, 1 µM or 5 µM). All media and cell culture reagents were purchased from Invitrogen (Carlsbad, CA, USA) unless otherwise stated. Both HS293 and HS346 were used in each of the experiments performed and no differences between these lines were observed.

### Aβ preparation and ThT fluorescence assay

To obtain fibrillar forms of Aβ_1–40_ and Aβ_1–42_, the Aβ peptides (Sigma, St. Louis, MO, USA) were dissolved in PBS and incubated with gentle shaking for 72 h at 37°C. Oligomeric Aβ species (AβO) were prepared by dissolving 1,1,1,3,3,3-hexafluoro-2-propanol (HFIP)-pretreated Aβ_1–40_ or Aβ_1–42_ (rPeptides, Bogart, GA, USA) in DMSO, followed by sonication and filtration. Aliquots were then stored at −80°C until time for usage.

Thioflavine T fluorescence assays were performed according to a previously described protocol [24]. Briefly, AβO_1–40_ (100 nM, 5 µM) or AβO_1–42_ (100 nM, 1 µM) were mixed with NPM, and thioflavine T and sodium azide (Sigma, St. Louis, MO, USA) were added to a final concentration of 1 µM and 0.02%, respectively. Thioflavine T fluorescence of samples was measured using an excitation wavelength of 450 nm and an emission wavelength of 485 nm at 37°C in 15 min intervals over a time period of 72 h using an Infinite M1000 plate reader (Tecan, Männedorf, Switzerland). To verify the aggregation forms of AβO in the experimental conditions used, HFIP-pretreated and sonicated Aβ_1–40_ and Aβ_1–42_ (100 nM, 5 µM and 100 nM, 1 µM, respectively) were incubated in NPM for 72 h at 37°C. Samples were analyzed by Western blotting as described by Stine [25]. Briefly, unheated samples in Laemilli buffer were separated by SDS/PAGE, blotted onto PVDF membranes and then blocked in 5% non-fat milk. The membranes were incubated at 4°C overnight with mouse anti-4G8 (1∶1000, Covance, Dedham, MA, USA), washed and then incubated with horseradish peroxidase-conjugated secondary antibody (donkey anti-mouse IgG; 1∶2000, Santa Cruz Biotechnology) for 1 h. Signals were visualized by incubation of the membranes in Enhanced Chemiluminescence (ECL) Plus reagents and exposure to Hyper Performance Chemiluminescence film (GE Healthcare, Buckinghamshire, UK).

### RNA isolation and real-time qPCR

RNA samples were isolated from hES cells differentiated for 28–35 days with TRI Reagent solution (Sigma, St. Louis, MO, USA) and 0.5 µg RNA was reverse transcribed by using a High Capacity RNA-to-cDNA Kit (Applied Biosystems, Foster City, CA, USA) (at 37°C for 60 min followed by 95°C for 5 min). Real-time qPCR was performed by using either SYBR Green I dye or TaqMan probe-based detection in a Step One Plus Real-Time PCR system (Applied Biosystems) according to the manufacturer's instructions. For the SYBR Green system, PCRs were performed at 95°C for 10 min, followed by 40 cycles at 95°C for 15 s and 60°C for 60 s. The specificity of each amplified product was confirmed using a dissociation curve analysis. For TaqMan probe-based detection, PCRs were performed at 50°C for 2 min, 95°C for 10 min, followed by 40 cycles at 95°C for 15 s and 60°C for 60 s. The TaqMan gene expression assays and primer sequences used in this study are listed in Table S1 and Table S2, respectively. All PCR reagents were purchased from Applied Biosystems. Cyclophilin A was used as an endogenous control and mRNA expression, relative to that in untreated cells (control), was quantified by the 2^-ΔΔCt^ method [23] using StepOne software v2.1 (Applied Biosystems). Prior to gene expression quantification, the primers were optimized to ensure close to 100% amplification efficiency.

### Immunocytochemistry

hES cells differentiated on poly-D-lysine- and laminin-coated cover slips were fixed with 4% paraformaldehyde at 4°C for 20 min, and thereafter permeabilized in blocking buffer, (3% normal donkey serum in PBS containing 0.05% Triton X-100) and incubated overnight at 4°C with the following antibodies; goat polyclonal IgG anti-human glutamic acid decarboxylase 67 (GAD-67; 1∶100, Santa Cruz Biotechnology, Heidelberg, Germany), goat polyclonal anti-human choline acetyltransferase (ChAT; 1∶100, Millipore, Temecula, CA, USA), mouse IgG2a monoclonal anti-human beta III-tubulin (1∶250, Abcam, Cambridge, UK), rabbit polyclonal anti-microtubule-associated protein 2 (MAP2) (1∶250, Millipore, Temecula, CA, USA) and rabbit polyclonal anti-human glial fibrillary acidic protein (GFAP; 1∶250, Dako, Glostrup, Denmark). Following 3×5 min washes in PBS, secondary antibodies conjugated with Alexa Fluor (AF 488 donkey anti-goat, AF 594 donkey anti-mouse or AF 488 donkey anti-rabbit, 1∶500; Molecular probes, Eugene, OR, USA) were added for a 1 h incubation period at RT in the dark. In addition, control experiments were performed where the primary antibody was substituted with blocking buffer to establish the specificity of the secondary antibodies. The cells were then washed with PBS (3×5 min) and mounted with Vectashield with DAPI for fluorescent microscopic analysis under a Nikon E800 microscope. Three to six random fields were counted in each experiment (>500 cells counted). Quantification was determined by counting the number of immunoreactive (ir) cells in each experiment divided by the total number of cells (DAPI-ir cells) in the same experiment.

### Cell proliferation assays

For proliferation assays, neurospheres from hES cells were dissociated with dispase (0,1 mg/ml) for 10 min at 37°C. The cells were plated in a 96-well tissue culture plate (25 000 cells/well) 24 h prior to the administration of NGF (50 ng/ml, Invitrogen) and oligomeric or fibrillar Aβ_1–40_ and Aβ_1–42_ (10 pM, 1 nM, 10 nM, 100 nM, 1 µM or 5 µM, rPeptide, Bogart, GA, USA or Sigma, St. Louis, MO, USA). Measurements of cell proliferation were performed after 14 days of NGF and Aβ treatment, using a Bromodeoxyuridine (5-bromo-2-deoxyuridine, BrdU) incorporation assay according to the manufacturer's instructions (Roche, Mannheim, Germany).

### Caspase 3/7 measurements

For caspase measurements, dissociated hES cell-derived neurospheres were plated in a 96-well black-walled tissue culture plate (20 000 cells/well) 24 h prior to administration of NGF (50 ng/ml, Invitrogen, Carlsbad, CA, USA), oligomeric or fibrillary Aβ_1–40_ or Aβ_1–42_ species (10 pM, 1 nM, 10 nM, 100 nM, 1 µM or 5 µM, rPeptide, Bogart, GA, USA). Caspase measurements were carried out 7 days after NGF and Aβ treatment using a Caspase-Glo 3/7 assay (Promega, Madison, USA) according to the manufacturer's instructions.

### Electrophysiological measurements of intracellular calcium

Human ES cells treated each week with NGF (50 ng/ml), AβO_1–40_ (100 nM) or AβO_1–42_ (1 µM) and differentiated for 28–35 days *in vitro* were maintained in culture medium and then loaded with the calcium-sensitive dye Fluo-3 AM (5 uM; Molecular Probes, Eugene, OR) for 60 min in Krebs-Ringer-HEPES (KRH) buffer (pH 7.4) containing pluronic acid (0.02%) and incubated at 37°C in the dark. The hES cell-derived neurons were stimulated with ACh (10 µM) and KCl (5 mM). Measurements of [Ca^2+^]i were also performed on untreated cells following acute exposure to oligomeric Aβ_1–40_ and Aβ_1–42_ (10 pM, 1 nM, 10 nM, 100 nM and 1 µM). The fluorescence measurements were performed using an Andor DU-860 electron multiplying (EM) CCD camera (Andor, Belfast, Ireland). To minimize the noise, EM gain was maintained at 10% and the CCD sensor was cooled to −80°C. The specimen was excited with a 470 nm LED array (Roithner Laser, Vienna, Austria) and the emission light was filtered with a 505 nm low-pass filter. Images were captured using custom-made software (‘KiaFluor’) that runs in Labview (National Instruments, USA). For each measurement, samples were exposed to light for 6 s and bleaching was negligible (below 5%) even after 10 exposure cycles. Kinetic imaging series were analyzed in Matlab (Mathworks, USA) following custom-made routines. Cells were considered to respond to the chemical stimuli if the mean fluorescence increased by >10% and if they also exhibited a fast, spontaneous calcium transient, characteristic for neurons [24]. The authors welcome any requests for software copies.

### Activation of the Akt cell-survival signaling pathway

To determine whether the effects of Aβ oligomers on neuronal differentiation were mediated by the involvement of phosphoinositide 3-kinase (PI3-K)-dependent activation of the Akt signaling pathway, hES cells that had differentiated for 28–35 days were stimulated with NGF (50 ng/ml) for 30 min or AβO_1–40_ (5 µM) or AβO_1–42_ (1 µM) for 60 min and then lysed in cold tris buffer saline (TBS) containing 1% Triton X-100, protease inhibitors and phosphatase inhibitors. Protein concentrations were determined using a DC protein assay kit (Biorad, Stockholm, Sweden). For inhibition of PI3-K signaling, cells were exposed to the inhibitor LY294002 (10 µM, Sigma) 5 h prior to stimulation with NGF or Aβ. Samples were denaturated and 20 µg protein was loaded and separated by SDS/PAGE and then blotted onto PVDF membranes and blocked in 5% bovine serum albumin. The membranes were incubated at 4°C overnight with primary antibodies: rabbit anti-phospho-Akt (1∶1000, Cell Signaling Technology), rabbit anti-Akt (1∶1000, Cell Signaling Technology, Danvers, MA, USA) and rabbit polyclonal anti-β-actin (1∶2000, Abcam). After washing, the membranes were incubated with horseradish peroxidase-conjugated secondary antibody (donkey anti-rabbit IgG or donkey anti-mouse IgG; 1∶2000, Santa Cruz Biotechnology). Signals were visualized by incubation of the membranes in ECL Plus reagents and exposure to Hyper performance Chemiluminescence film. The films were scanned and the optical density of each band, normalized to that of β-actin, was analyzed by using the public domain National Institutes of Health Image J software.

### Statistical analysis

Results are expressed as mean ± SD from 3–4 independent experiments and analyzed by means of unpaired Student's *t*-test, the Z-test for differences in proportion, or one-way ANOVA followed by Dunnett's *post hoc* test, as appropriate using GraphPad Prism 5.0 (GraphPad Software, Inc. La Jolla, CA, USA). Results from real time qPCR measurements are expressed as mean fold change ± S.E. fold change and the ΔΔCt-values from 3–6 independent experiments were analyzed by means of unpaired Student's *t*-test (GraphPad Prism 5.0). Values of *p*<0.05 were considered significant.

## Results

### Treatment of hES cells with nerve growth factor promotes differentiation of basal forebrain cholinergic neurons

**Figure 1:**
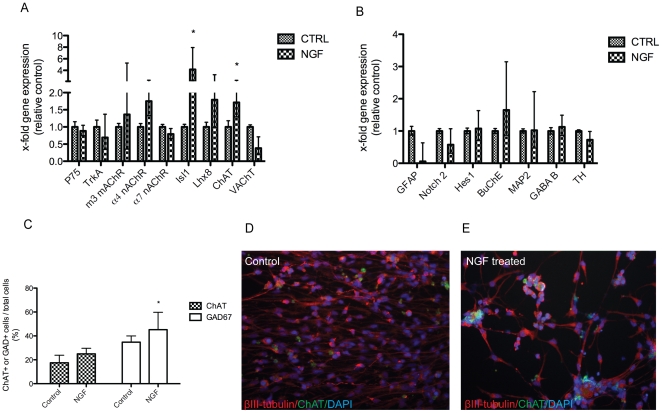
NGF promotes the differentiation of hES cells into cholinergic and GABAergic neurons. Expression of neuronal and glial markers following NGF (50 ng/ml) treatment in hES cells differentiated for 28–35 days *in vitro*. (A, B) Gene expression levels were assessed by real-time qPCR and normalized to those of cyclophilin. Values are expressed as mean fold change (± S.E.) from 3–6 independent experiments. *p<0.05 compared with controls (unpaired Student's *t*-test). (C) The proportion of cells expressing the choline acetylcholine transferase (ChAT) or glutamic acid decarboxylase GAD67 following treatment with NGF. Values are expressed as mean ± SD (>500 cells counted). *p<0.05 compared with controls (unpaired Student's *t*-test). (D) Immunostaining of ChAT (green) and neuronal marker βIII-tubulin (red) in untreated cells and in (E) NGF-differentiated hES cells (at 20x). Nuclei were stained with DAPI (blue).

The gene expression for glial and neuronal markers with emphasis on the cholinergic system was assessed after treatment with NGF in hES cell-derived neurons by real time qPCR and quantified relative to control (untreated) cells using the ΔΔCt method [23]. Following 28–35 days of differentiation, hES cells expressed glial fibrillary acidic protein (GFAP), the neuronal suppressor genes Notch2 and hairy and enhancer of split (Hes1) as well as the late neuronal marker microtubule-associated protein 2 (MAP2) (Figure 1). Furthermore, the differentiated cells expressed transcripts for the cholinergic receptors p75 neurotrophic receptor (p75^NTR^), neurotrophic tyrosine kinase receptor type 1 (TrkA), the α4 and α7 nicotinic acetylcholine receptor (nAChR) and the M3 muscarinic acetylcholine receptor (mAChR) (Figure 1). In addition, we detected expression of the cholinergic enzymes choline acetyltransferase (ChAT) and vesicular acetylcholine transporter (VAChT) (Figure 1). Cells exposed to NGF during differentiation showed an increased gene expression of ChAT (1.7-fold increase, *p*<0.05).

In order to further investigate cholinergic characteristics of the hES cell-derived neurons, we examined the number of ChAT^+^ cells co-expressed with the neuronal marker βIII-tubulin^+^ cells by using immunocytochemistry (Figure 1C–E). Following NGF treatment, the proportion of neurons expressing ChAT was increased (25.0±4.7%) compared with untreated cells (17.5±6.4%) (Figure 1C). A significant increase in the number of glutamic acid decarboxylase GAD67^+^ cells was also observed in NGF treated hES cells (45.2±14.6%) compared with untreated cells (34.8±5.2%, *p*<0.05) (Figure 1C).

### Basal forebrain identity of cholinergic hES cell-derived neurons

To characterize the subregional identity of hES cells treated with NGF, we analyzed the expression of transcription factors known to play a key role in regulating the development of BFCNs at 0, 3, 9, 20 and 28 days of differentiation. We observed a high expression of the transcription factor Nk2-homebox 1 (Nkx2-1) that decreased during 28 days of differentiation. The expression of the LIM-homebox gene, Lhx8 increased as hES differentiation progressed (Figure S2). The cholinergic phenotype of the derived neurons was also confirmed by an increased ChAT expression after NGF exposure during differentiation, while a decreased expression of ChAT was observed in the untreated cells (Figure S2). Interestingly, the peak in Lhx8 expression at 9 days of differentiation coincided with the increase in ChAT detection in NGF treated cells but not in control cells, suggesting that NGF is an important inductive factor for promoting the development of BFCNs. Furthermore, exposure of hES cells to NGF during 28 days of differentiation significantly increased (4.2-fold, *p*<0.05) the expression of the LIM-homeobox gene Islet-1 (*Isl1*) (Figure 1). Surprisingly, transcripts for ChAT, Lhx8 and Nkx2-1 were also detected at day 0 of differentiation, and at later stages of differentiation in the absence of NGF, suggesting a default induction for hES cells to differentiate into telencephalic neurons (Figure S2).

### Effects of oligomeric Aβ_1–40_ and Aβ_1–42_ on hES cell differentiation

The permanence of oligomeric Aβ species throughout the experiments was assessed by means of a thioflavine T fluorescence assay (Figure S3A). Western blotting analysis was also performed as previously described [25], which confirmed that HFIP-pretreated Aβ_1–40_ and Aβ_1–42_ was converted from monomers to oligomeric species during our experimental conditions (Figure S3B).

**Figure 2:**
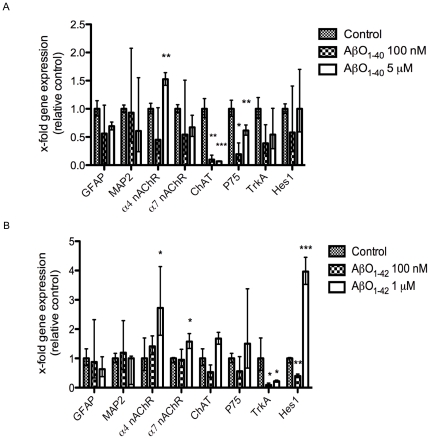
Gene expression of hES cells exposed to Aβ_1–40_ and Aβ_1–42_ oligomers. Expression of neuronal and glial markers following (A) AβO_1–40_ (100 nM or 5 µM) and (B) AβO_1–42_ (100 nM or 1 µM) treatment of hES cells differentiated 28–35 days *in vitro*. Values are expressed as mean fold change (± S.E.) from 3–6 independent experiments. *p<0.05, **p<0.01, ***p<0.001 (unpaired Student's *t*-test).

**Figure 3:**
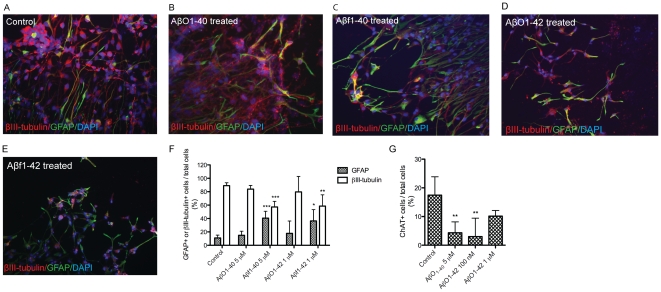
Fibrillar Aβ_1–40_ and Aβ_1–42_ induces glial differentiation of hES cells. Immunocytochemical staining for neuronal and glial markers following NGF, AβO or Aβf exposure in hES cells differentiated for 28–35 days *in vitro*. (A) Immuno- reactivity for βIII-tubulin (red) and glial fibrillary acidic protein, GFAP (green) in untreated cells, (B) AβO_1–40_ (5 µM) exposed hES cells, (C) Aβf_1–40_ (5μM) exposed hES cells, (D) AβO_1–42_ (1 µM) exposed hES cells and (E) Aβf_1–42_ (1 µM) exposed cells (at 20x). Nuclei were stained with DAPI (blue). (F) The proportion of cells expressing βIII-tubulin or GFAP, following Aβ treatment. Fibrillar Aβ_1–40_ (5 µM) and Aβ_1–42_ (1 µM) decreased the expression of βIII-tubulin. (G) ChAT^+^ cells following AβO_1–40_ (5 µM) or Aβ_1–42_ (100 nM or 1 µM) exposure (>500 cells counted). Values are expressed as mean ± SD from three independent experiments. *p<0.05, **p<0.01 and ***p<0.001 compared with controls (unpaired Student's *t*-test).

To study the effects of oligomeric Aβ (AβO) on hES cell cholinergic neuronal differentiation we examined the gene expression profile following exposure to AβO_1–40_ (100 nM, 5 µM) and AβO_1–42_ (100 nM, 1 µM). A high concentration of Aβ_1–42_ (5 µM) was toxic to the cells, affecting their morphology and treatment at this high concentration was discontinued (data not shown). Exposure to AβO_1–40_ (5 µM) revealed a significant increase in the expression of the α4 nAChR subunit (1.6-fold, *p*<0.05). There was also a significant decrease in expression of ChAT (*p*<0.01 and *p* <0.001 for 100 nM and 5 µM, respectively) and the neurotrophin receptor p75^NTR^ (*p*<0.05 and *p*<0.01 for 100 nM and 5 µM, respectively) following Aβ_1–40_ treatment (Figure 2A). Immunocytochemical analyses of the number of βIII-tubulin^+^ cells following AβO_1–40_ (5 µM) exposure (84.0±5.5%) were similar to those in untreated cells (89.0±4.3%) (Figure 3F). Similarly, the proportion of GFAP^+^ cells did not differ after AβO_1–40_ exposure (15.0±6.1%) compared with untreated cells (11.0±4.3%) (Figure 3F). However, we found a significant decrease in the number of ChAT^+^ cells following exposure to Aβ_1–40_ (5 µM) (4.3±3.8%, *p*<0.05) compared with untreated cells (17.5±6.4%) (Figure 3G), that correlated with a decrease in ChAT gene expression.

Treatment with AβO_1–42_ (1 µM) resulted in a significant increase in gene expression of the α4 nAChR (2.7-fold, *p*<0.05) and α7 nAChR (1.6-fold *p*<0.05) subunits as well as a significant decrease in gene expression of the tyrosine kinase receptor (TrkA) (*p*<0.05 for both 100 nM and 1 µM) (Figure 2B). Immunocytochemical staining revealed that AβO_1–42_ (1 µM) treatment did not alter neither the proportion of βIII-tubulin^+^ cells (79.8±23.0%) nor the proportion of GFAP^+^ cells (17.9±18.4%) compared with untreated cells (89.0%±4.3% and 11.0±4.3% for βIII-tubulin^+^ and GFAP^+^ cells, respectively) (Figure 3F), but significantly decreased the number of MAP2^+^ cells (11.5±0.7%, *p*<0.01) compared with untreated cells (25.1±1.0) (Figure S4). Further, a decrease in the number of ChAT^+^ cells was observed in cells treated with AβO_1–42_ (100 nM) (3.0±6.4%, *p*<0.01) as well as AβO_1–42_ (1 µM) (10.1±2.0%, *p*>0.05) compared with untreated cells (17.5±6.4%) (Figure 3G).

### Effects of fibrillar Aβ_1–40_ and Aβ_1–42_ on hES cell differentiation

**Figure 4:**
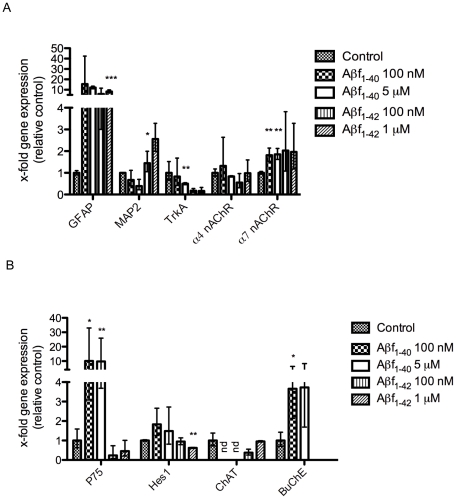
Gene expression of hES cells exposed to fibrillar Aβ_1–40_ and Aβ_1–42_. Expression of neuronal and glial markers, following Aβf_1–40_ (100 nM or 5 µM) and Aβf_1–42_ (100 nM or 1 µM) treatment in hES cells differentiated for 28–35 days *in vitro* (A, B). Values are expressed as mean fold change (± S.E fold change), from 3–6 independent experiments, *p<0.05, **p<0.01, ***p<0.001 (unpaired Student's *t*-test).

Next, we examined the effects of fibrillar Aβ (Aβf) (100 nM, 5 µM of Aβ_1–40_) and (100 nM, 1 µM of Aβ_1–42_) on hES cell differentiation. The hES cells exposed to Aβf_1–40_ (Aβ 100 nM and 5 µM, respectively) demonstrated an increased gene expression of p75^NTR^ (10.1-fold, *p*<0.05, 9.1-fold, *p*<0.01, respectively), GFAP (15.4-fold and 12.1-fold, *p*>0.05, respectively), butyrylcholine esterase (BuChE) (3.7-fold, *p*<0.05, respectively), and Hes1 (1.8-fold and 1.5-fold, *p*>0.05, respectively) (Figure 4). In addition, treatment with Aβf_1–40_ resulted in a significant increase in α7 nAChR subunit gene expression (1.8-fold, *p*<0.01, for both 100 nM and 5 µM), and a significant decrease in TrkA (*p*<0.01, 5 µM) gene expression (Figure 4). Interestingly, no ChAT gene expression was detected following Aβf_1–40_ (100 nM, 5 µM) exposure (Figure 4B). Consistent with the real time qPCR data, the proportion of GFAP^+^ cells increased following Aβf_1–40_ (5 µM) exposure (40.0±10.3%, *p*<0.001) compared with untreated cells (11.0±2.5%), while in contrast, a decrease in the number of βIII-tubulin^+^ cells (57.0±8.5%, *p*<0.001) was observed in comparison with untreated cells (89.0±2.5%) (Figure 3F).

The analysis of gene expression in cells treated with Aβf_1–42_ revealed significant increases in both GFAP (8.3-fold, *p*<0.001, Aβ 1 µM) and MAP2 (1.4-fold, *p*<0.05, Aβ 100 nM) and a slight increase for the α7 nAChR transcript (2-fold, *p*>0.05, Aβ 100 nM and 1 µM) (Figure 4A). The observed increase in GFAP gene expression was in line with the observation of a significant increase in the proportion of GFAP^+^ cells (36.3±17.1%, *p*<0.05) following Aβf_1–42_ (1 µM) exposure compared with untreated cells (11.0±4.3%), whereas a significant decrease in βIII-tubulin^+^ cells (58.5±16.8% *p*<0.01) was observed compared with control (89.0±4.3%) (Figure 3F).

### Effects of fibrillar and oligomeric Aβ_1–40_ and Aβ_1–42_ on hES cell proliferation

β-amyloid has previously been reported to have differential effects on hES cell proliferation depending on the aggregation state of the peptide [26]. We therefore investigated if any of the Aβ species studied here were mitogenic by exposing the cells to fibrillar or oligomeric Aβ_1–40_ and Aβ_1–42_ for 14 days *in vitro*, and thereafter used a cell proliferation colorimetric assay to measure BrdU incorporation. AβO_1–40_ treatment increased the cell proliferation significantly at 100 nM and 5 µM concentrations (*p*<0.001 and p<0.05, respectively), compared to untreated cells (Figure S5A). There was no significant increase in proliferation observed following treatment with neither AβO_1–42_, Aβf_1–40_ nor Aβf_1–42_ (Figure S5).

### Caspase 3 and 7 measurements following exposure to oligomeric and fibrillar Aβ

To investigate whether exposure of hES cells to Aβ could lead to activation of downstream apoptotic signaling events, caspase 3/7 activity measurements were carried out. We observed that only low concentrations (100 nM) of Aβf_1–40_, AβO_1–42_ and Aβf_1–42_ increased caspase 3/7 activity significantly (*p*<0.01, *p*<0.001, *p*<0.05, respectively) after 7 days exposure (Figure S5). No significant changes in caspase 3/7 activity were observed in hES cells exposed to AβO_1–40_ (Figure S5).

### Characterization of functional properties of hES cell-derived neurons

**Figure 5:**
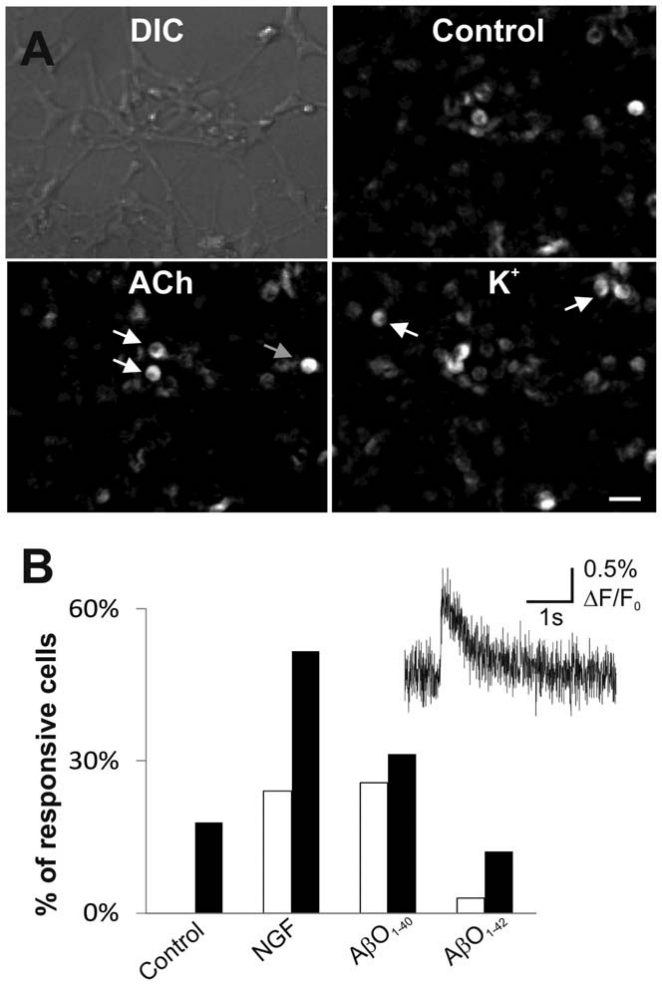
Characterization of functional properties of hES cell-derived neurons. Fluorescence imaging with the calcium indicator Fluo-3 in hES cell-derived neurons, differentiated for 28 days *in vitro*. (A) The top-left panel is a differential interference contrast (DIC) photomicrograph showing all the cells in a field of oligomeric Aβ_1–40_ (100 nM) exposed hES cells. Fluorescence images of cell bodies in control conditions (top-right), 5 min after ACh (10 µM) (bottom-left), and 5 min after the addition of 5 mM KCl (bottom-right). Images are shown with arbitrary pixel intensity (0 = 100% black and 255 = 100% white), scale bar = 50 µm. Arrows indicate examples of cell bodies that showed fluorescence changes of >10% ΔF/F_0_. (B) Summary showing the percentages of cell bodies that reacted with more than a 10% ΔF/F_0_ increase in fluorescence in response to ACh (10 µM) (white bars) and 5 mM KCl (black bars), with different pre-conditions (control, NGF (50 ng/ml), AβO_1–40_ (100 nM) and AβO_1–42_ (1 µM)). *Inset* shows an example of a fast spontaneous calcium transient recorded in a neuron from NGF (50 ng/ml) treated hES cells in the presence of ACh (10 µM).

Functional properties of the hES cell-derived neurons were evaluated by [Ca^2+^]_i_ electrophysiological recordings (bulk loading cultures with Fluo-3 indicator). Cells were considered to respond to the stimuli if there was an increase in fluorescence (ΔF/F_0_) of >10%. All cells that responded to the stimuli (>10% increase in fluorescence) also exhibited a fast, spontaneous calcium transient, characteristic for neurons (representative example shown in Figure 5B) [24]. ACh (10 µM) did not evoke any [Ca^2+^]_i_ increase in the untreated cells. We observed an increase in [Ca^2+^]_i_ following depolarization of the cells with KCl (5 mM) in 17.9% of the untreated cells (Figure 5). The hES cell-derived neurons exposed to NGF during differentiation exhibited significant increases in the number of cells responding to both ACh (24.1%, *p*<0.01) and KCl (51.7%, *p*<0.01) compared with untreated cells, reflecting an increase in the proportion of neurons expressing cholinergic receptors as well as voltage-gated Ca^2+^ channels (VGCCs) (Figure 5).

To investigate immediate effects of Aβ on [Ca^2+^]_i_ in hES cell-derived neuronal populations, we exposed untreated cells which had differentiated for 28–35 days *in vitro*, to oligomeric Aβ_1–40_ as well as to Aβ_1–42_. The acute application of either AβO_1–40_ or AβO_1–42_ (10 pM, 1 nM, 10 nM, 100 nM and 1 µM) to hES cells failed to evoke a >10% increase in fluorescence in our Ca^2+^-imaging experiments (data not shown). The presence of functional cholinergic receptors and VGCCs on neuronal cells derived from hES cells treated with AβO_1–40_ and AβO_1–42_ during 28–35 days of differentiation was also evaluated. In cells exposed to AβO_1–40_, the proportion of cells responding to ACh (25.7%) was significantly increased (*p*<0.01) compared with untreated cells (Figure 5). Depolarization with KCl (5 mM) increased [Ca^2+^]_i_ in a larger population of hES-derived neuronal cells exposed to AβO_1–40_ (31.4%) compared with controls (17.9%) (Figure 5). In contrast, in the AβO_1–42_-treated cells, the proportion of cells responding to KCl (12.1%) was decreased compared with controls (17.9%). However, a small proportion of these cells did respond to ACh (3.0%), which was not detected in the untreated cells (Figure 5).

### NGF promotes cholinergic neuronal differentiation through the PI3-K/Akt pathway

**Figure 6:**
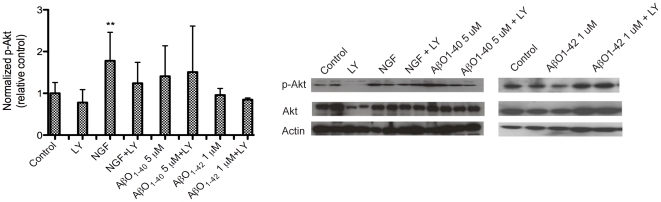
NGF-induced phosphorylation of Akt is mediated through the PI3-K signaling pathway. To measure the levels of phosphorylated Akt (p-Akt) mediated through stimulation of the PI3-K signaling pathway, hES cells were treated with the PI3-K inhibitor LY294002 (10 µM) 5 h prior to stimulation with NGF (50 ng/ml) or AβO_1–40_ (5 µM). Cell lysates were analyzed by Western blotting, using phospho-specific antibodies, and β-actin served as a loading control. (A) Following stimulation with NGF or AβO_1–40_, p-Akt levels were 1.74±0.74 and 1.66±1.54 relative to controls, respectively. Induction of p-Akt by NGF was inhibited by LY294002. However, the AβO_1–40_-dependent phosphorylation of Akt was not inhibited by LY294002. Values are expressed as mean ± SD (relative to controls), *p<0.05 (unpaired Student's *t*-test). (B) Representative immunoblots of p-Akt, total Akt and β-actin.

To examine the signaling mechanisms involved during hES cell differentiation following NGF and AβO exposure, Akt phosporylation was analyzed since the phosphoinositide-3-kinase (PI3-K/Akt) signaling pathway has been implicated in promoting neuronal survival and differentiation [27]. Differentiated hES cells stimulated with NGF (50 ng/ml) or AβO_1–40_ (5 µM) induced phosphorylation of Akt (Figure 6). NGF induced the highest p-Akt expression (1.74±0.74 relative to controls, *p*<0.01) that was inhibited in the presence of the PI3-K inhibitor LY294002 (10 µM) (Figure 6). On the other hand, the increased expression of p-Akt (1.66±1.54 relative to controls), following exposure to AβO_1–40_ (p>0.05) was not inhibited by LY294002 (Figure 6). Cells stimulated with AβO_1–42_ (1 µM) did not induce any significant changes in p-Akt expression (0.95±0.16) (Figure 6).

## Discussion

Elucidation of the molecular signals governing cholinergic development and dysfunction is essential for the design of novel drugs that could stimulate regeneration and support neuroprotective mechanisms in the brain of patients afflicted with AD.

The generation of new neurons in the adult brain may be associated with the rewiring of the brain and regulation of cognitive functions and these processes are most likely influenced by disease pathology. Therefore, a further understanding of how AD-related physiological and pathological conditions alters the behavior of stem cell reservoirs residing in neurogenic regions in the brain is crucial for the implementation of future regenerative strategies.

BFCNs are dependent on NGF for the maintenance of their cholinergic phenotype and their cholinergic synaptic integrity [6]. NGF has been shown to regulate dendritic growth, axon guidance, long-term potentiation (LTP), synaptic plasticity and neurotransmitter release [28]. An imbalance in NGF axonal transport and loss of its high-affinity receptor TrkA has been observed in both early and late stages of AD [29]. In clinical studies, intracerebroventricular NGF administration in AD patients demonstrated an increase in the number of ^11^C-nicotine binding sites measured by PET [30] and a reduced rate of cognitive decline [31]. We recently reported that neurotrophic factors, including NGF, modulate the fate of hES cells and promote the differentiation into BFCNs [12].

In the present study, we further characterized and quantified the expression of neuronal and cholinergic markers following exposure of hES cells to NGF during their differentiation by means of quantitative gene expression profiling and fluorescent immunocytochemistry. Here, we report that NGF treatment induces an increase in the number of neurons expressing a cholinergic phenotype in agreement with our previous observations [12]. Furthermore, we observed that NGF influences neuronal phenotypic expression in hES cultures and gives rise to both cholinergic as well as GABAergic neurons. Intriguingly, transcripts for the homeobox genes Nkx2-1, and Lhx8 as well as ChAT were observed at day 0 of differentiation, suggesting a default forebrain identity of the two lines of hES cells used in this study. These observations concur with an earlier study reporting the expression of a cholinergic system in murine stem cells at a very early stage during embryonic development [32]. It has been suggested that the early expression of a cholinergic system in both neuronal and non-neuronal cells is indicative of a regulatory function of this system, that mediates basic cellular functions like proliferation, gene expression, differentiation, cytoskeletal mobility, migration, and secretion [32]. Considering these many different roles, it is likely that the cholinergic system will not function in the same manner throughout embryonic development, taking over its main role within the nervous system only at later stages.

The PI3-K/Akt pathway has been implicated in promoting neuronal survival, proliferation and differentiation, synaptogenesis as well as translocation of VGCCs to the plasma membrane. It has earlier been reported that NGF induces the expression of cholinergic markers via the PI3-K/Akt pathway [33]. Here, we demonstrate that NGF induced an increase in phospho-Akt, which was inhibited in the presence of the PI3-K inhibitor LY294002. Thus, these findings indicate that NGF mediates differentiation and cell survival in hES cells by activating this pathway. Furthermore, we showed that NGF significantly increases the number of functional neurons expressing cholinergic receptors and VGCCs, indicating that NGF is required not only for maintaining a cholinergic phenotype, but also for the maturation of newly differentiated neurons.

The pathophysiological environment in the AD brain may present limitations for the use of therapies intending to stimulate neurogenesis, and we have earlier proposed that modulation of the microenvironment with disease-modifying drugs may augment neuroregenerative processes [34]. PET amyloid imaging studies in patients with mild cognitive impairment and mild AD have demonstrated that the deposition of fibrillar Aβ is one of the early initiating pathogenic events in the disease process [35]. However, in recent years, the focus has shifted towards oligomeric forms of Aβ which are also present in the AD brain. These soluble forms of Aβ are suggested to be the most toxic, as they block LTP in hippocampal slices, impair synaptic calcium homeostasis and induce cell death in cultured neurons [8], [36]. A previous study demonstrated that hES cells transplanted into the developing mouse brain can differentiate into neural lineages and generate mature, functional human neurons that successfully integrate into the adult mouse forebrain [37]. Although these findings hold great promise for cell-replacement therapy, not much is known about how amyloid pathology in AD may influence the differentiation and function of stem cell-derived neurons. To address this, we systematically investigated the effects of both oligomeric and fibrillar forms of Aβ_1–40_ and Aβ_1–42_ on hES cell-derived neuronal populations *in vitro*, as these may have relevance with regards to synaptic plasticity in the brain. A dose-dependent increase in proliferation as measured by BrdU incorporation was observed in hES cells treated with oligomeric and fibrillar Aβ_1–40_ and Aβ_1–42_. Surprisingly, immunocytochemical staining revealed the presence of >75% βIII-tubulin^+^ cells in cultures exposed to Aβ_1–42_ and Aβ_1–40_ oligomers, a proportion that did not differ from that of untreated cells. However, the number of MAP2 positive cells (late neuronal marker) was significantly decreased following oligomeric Aβ_1–42_ exposure, suggesting a decrease in the capacity of these cells to differentiate into mature neurons.

It has earlier been postulated that Aβ may transiently promote the generation of nonfunctional neurons from neural stem cells [38]. In the present study, we determined whether the hES cell-derived neurons exhibited functional cholinergic receptors and VGCCs. We found that Aβ_1–40_ oligomers increased the number of functional neurons but suppressed cholinergic neuronal differentiation, whereas in contrast, Aβ_1–42_ oligomers decreased the proportion of functional neurons as well as suppressed neuronal cholinergic differentiation. The impaired functional response was not due to apoptotic events since no significant increase in caspase 3/7 activity was detected at similar concentrations. Moreover, we demonstrated that both fibrillar

Aβ_1–40_ and Aβ_1–42_ promote glial differentiation in hES cells, since significant increases in the expression of glial markers GFAP and the neuronal suppressor gene Hes1 were detected. An increased α7 nAChR expression was also observed in hES cells treated with fibrillar Aβ_1–40_, which most likely reflects an up-regulation of α7 nAChRs on astrocytes in response to increased amyloid deposition in AD, in concurrence with a previous observation in autopsy brain tissue from AD patients [39].

The presence of nAChRs and mAChRs are not exclusively located on cholinergic neurons, but these are also expressed on several other neuronal subtypes [40]. Thus, this could account for the increase in the number of neurons responding to ACh following oligomeric Aβ_1–40_ exposure. Aβ may also have a physiological role in the brain and we suggest that it is also possible that oligomeric Aβ_1–40_ augments the number of functional cholinergic receptors as a compensatory mechanism, whereas Aβ_1–42_ oligomers may specifically promote differentiation of hES cells to non-functional neurons. Previous studies investigating the role of Aβ on neurogenesis have provided conflicting results. Data in support of both enhanced neurogenesis [16], [17] as well as neurotoxic effects [41] have been reported. The different experimental conditions applied in these earlier reported studies and our present study, including the aggregation states of Aβ as well as the different source of stem cells (fetal or adult as well as human or murine) could account for the discrepant findings.

Since oligomeric Aβ did not alter the number of βIII-tubulin positive cells, we investigated whether oligomeric Aβ exposure stimulated PI3-K/Akt signaling, since this pathway is implicated in promoting neuronal survival and differentiation. We observed an increase in protein levels of phospho-Akt following exposure to Aβ_1–40_ oligomers, which was not inhibited by LY294002. These results suggest that the Aβ_1–40_-induced phosphorylation of Akt in hES cell-derived neurons is not mediated by the PI3-K pathway and occurs through yet unknown mechanisms. A recent study reported that intracellular Aβ_1–42_ oligomers inhibit PI3-K/Akt signaling in neuronal cells in culture [42]. However, we did not observe any change in phospho-Akt following stimulation with Aβ_1–42_ oligomers in our present study.

In conclusion, our current findings indicate that factors governing neurogenesis in the brain depend on the state of aggregation and the concentration of Aβ species. In addition, our data supports the idea that latent progenitor reservoirs existing in neurogenic regions of the brain have a diminished capacity for supporting neurogenesis in AD. This may result from reduced concentrations of trophic factors, impaired trophic metabolic signaling and as a consequence of Aβ oligomers transiently promoting the generation of immature neurons, while Aβ fibrillar deposits may promote the differentiation towards cell types associated with inflammatory lesions. Efforts to boost the brain with trophic support as well as the development of inhibitors that target Aβ-induced signaling pathways or specific aggregation forms of Aβ, in particular Aβ_1–42_ oligomers, can thus promote the survival and maturation of functional stem cell-derived neurons in AD.

## Supporting Information

Figure S1 Characterization of hES cells.
(TIF)

Figure S2 Gene expression of hES cells during differentiation.
(TIF)

Figure S3 Oligomeric Aβ does not fibrillize in the conditions under which hES cells were cultured.
(TIF)

Figure S4 Oligomeric Aβ_1-42_ decreases the number of MAP2 positive neurons.
(TIF)

Figure S5 Cell proliferation and caspase activity in hES cells treated with fibrillar and oligomeric Aβ_1-40_ and Aβ_1-42_.
(TIF)

Table S1 TaqMan Gene Expression Assay ID.
(DOC)

Table S2 Primer sequences used in real-time quantitative PCR.
(DOC)
