# Supplementary figures and images for: β-Amyloid 1-42 Oligomers Impair Function of Human Embryonic Stem Cell-Derived Forebrain Cholinergic Neurons
Source: PLoS One. 2010 Dec 17;5(12):e15600. doi: 10.1371/journal.pone.0015600 (PMC3003688; doi:10.1371/journal.pone.0015600)

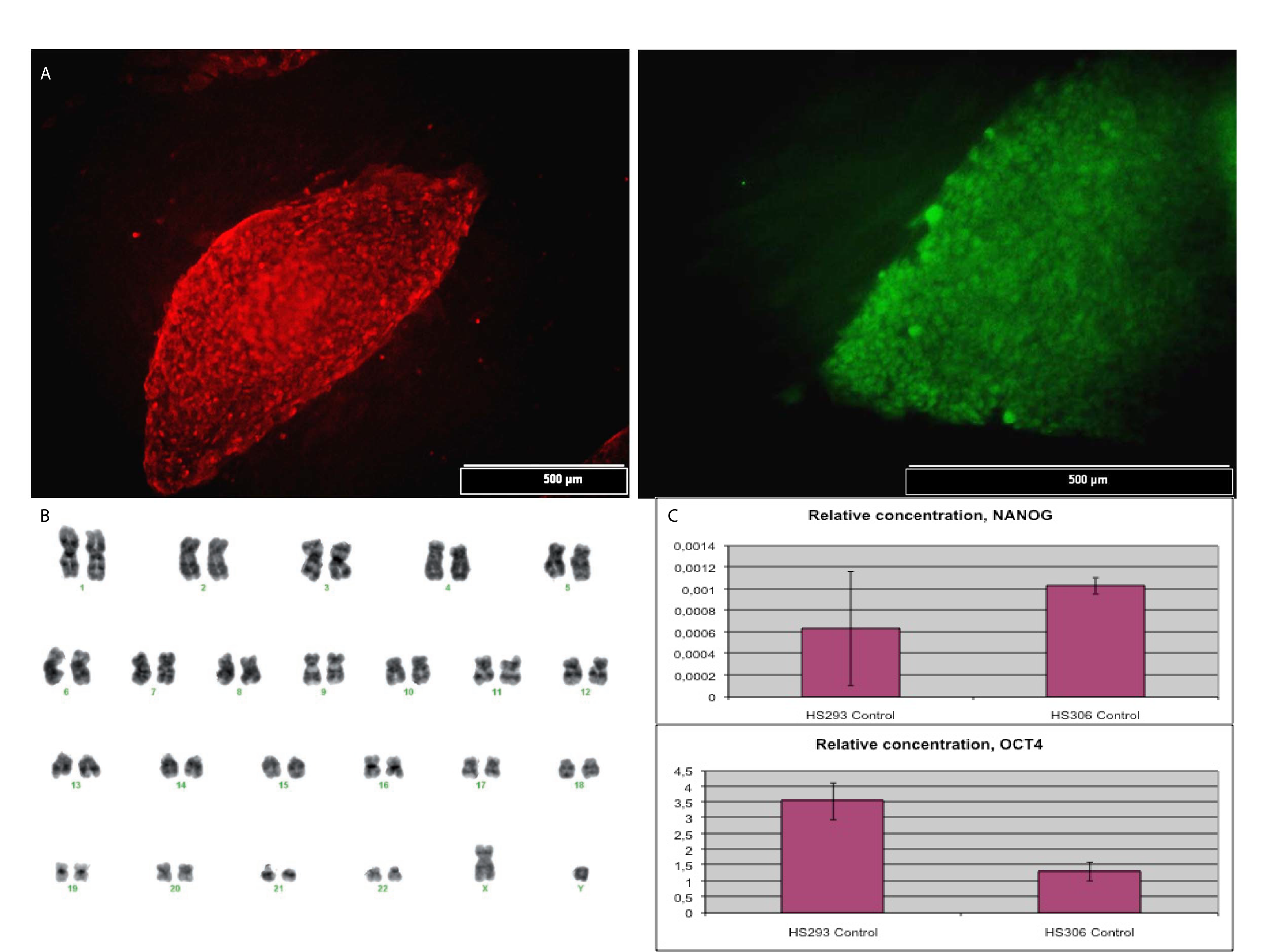

Supplement: Figure S1 — Characterization of hES cells. (TIF) [file pone.0015600.s001.tif]

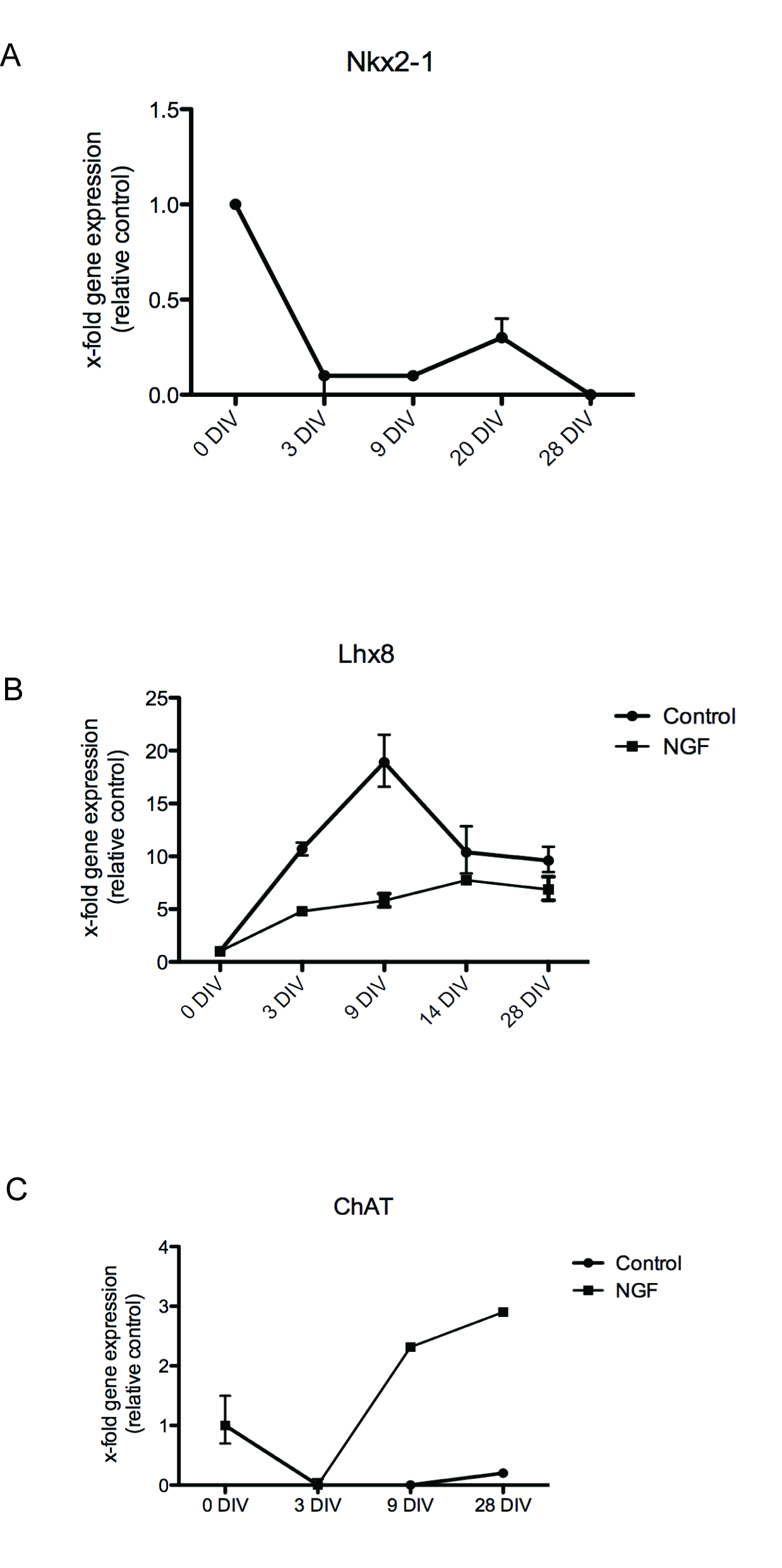

Supplement: Figure S2 — Gene expression of hES cells during differentiation. (TIF) [file pone.0015600.s002.tif]

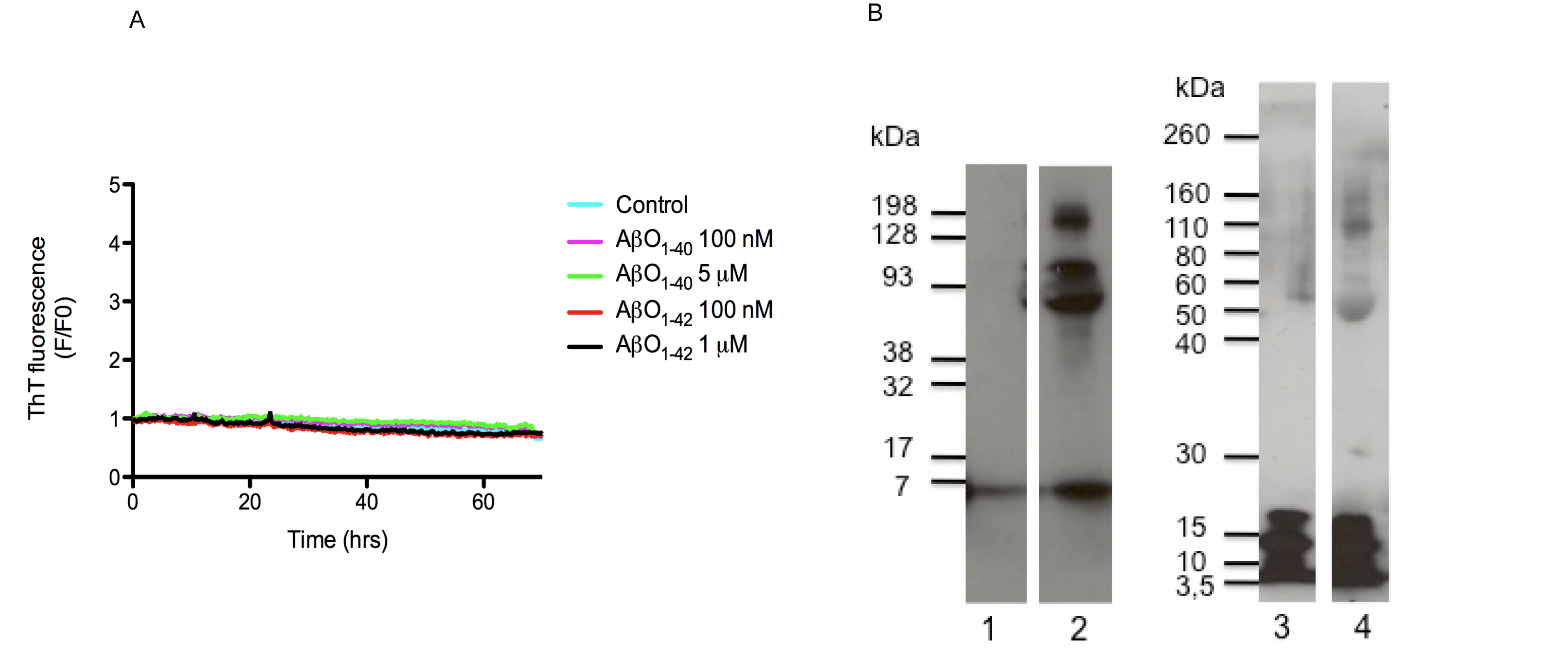

Supplement: Figure S3 — Oligomeric Aβ does not fibrillize in the conditions under which hES cells were cultured. (TIF) [file pone.0015600.s003.tif]

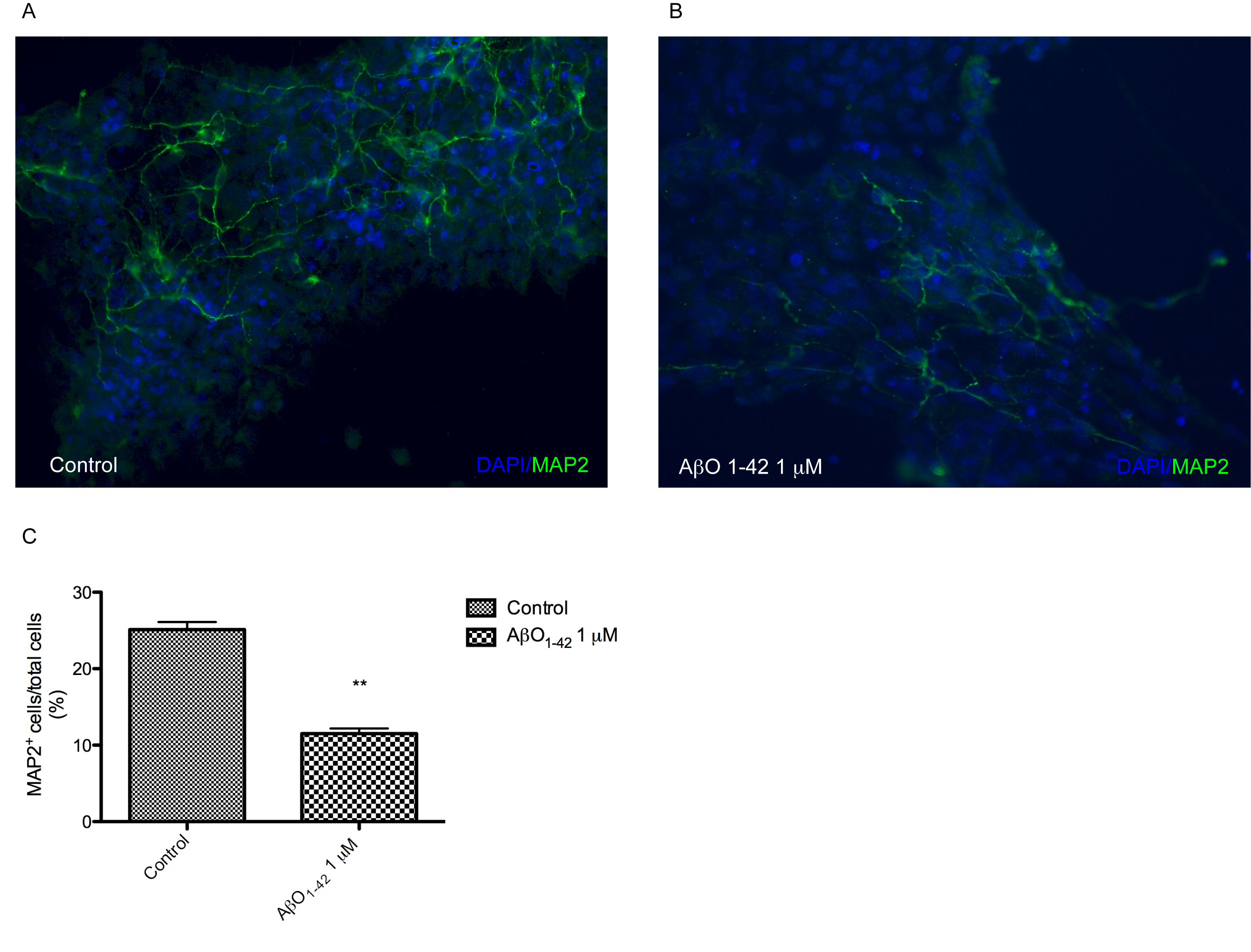

Supplement: Figure S4 — Oligomeric Aβ1-42 decreases the number of MAP2 positive neurons. (TIF) [file pone.0015600.s004.tif]

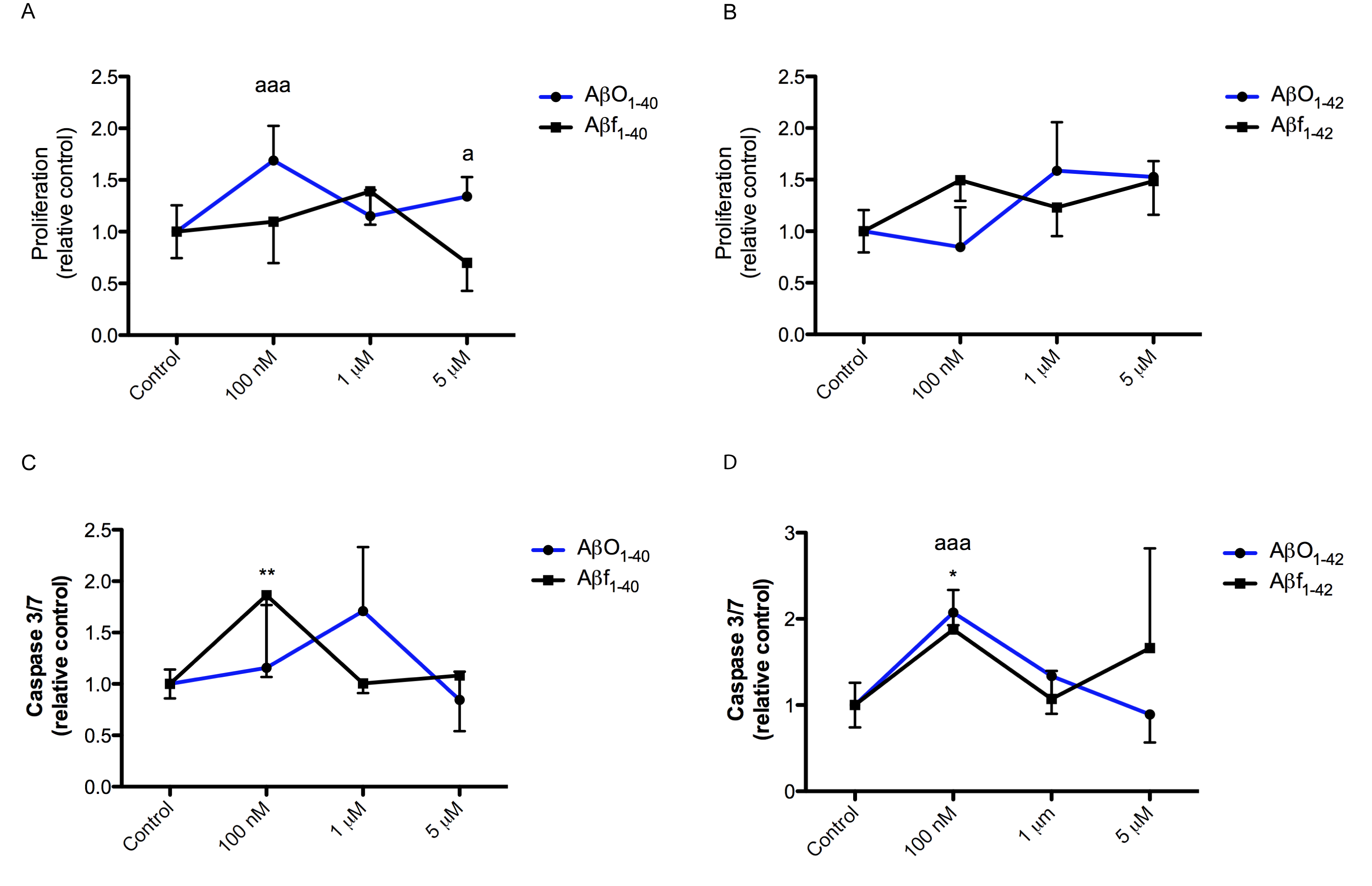

Supplement: Figure S5 — Cell proliferation and caspase activity in hES cells treated with fibrillar and oligomeric Aβ1-40 and Aβ1-42. (TIF) [file pone.0015600.s005.tif]
