# Supplementary material for: β-Amyloid 1-42 Oligomers Impair Function of Human Embryonic Stem Cell-Derived Forebrain Cholinergic Neurons
Source: PLoS One. 2010 Dec 17;5(12):e15600. doi: 10.1371/journal.pone.0015600 (PMC3003688; doi:10.1371/journal.pone.0015600)
Supplement: Table S1 — TaqMan Gene Expression Assay ID. (DOC) [file pone.0015600.s006.doc]

**Supplementary Table 1. TaqMan Gene Expression Assay ID.**

| **Target Gene** | **TaqMan Gene Expression Assay ID** | **Product size (bp)** |
| --- | --- | --- |
| Neurotrophic tyrosine kinase, receptor, type 1 (TrkA) | Hs00176787_m1 | 68 |
| NK2 homeobox 1 (Nkx2-1) | Hs00163037_m1 | 96 |
| Choline Acetyltransferase (ChAT) | Hs00252848_m1 | 64 |
| LIM homeobox 8 (Lhx8) | Hs00418293_m1 | 66 |
| Glial fibrillary acidic protein (GFAP) | Hs00157674_m1 | 75 |
| Nerve growth factor receptor (p75^NTR^) | Hs00182120_m1 | 69 |
| Human PPIA (cyclophilin A) | 4326316E | 98 |
