# Supplementary material for: β-Amyloid 1-42 Oligomers Impair Function of Human Embryonic Stem Cell-Derived Forebrain Cholinergic Neurons
Source: PLoS One. 2010 Dec 17;5(12):e15600. doi: 10.1371/journal.pone.0015600 (PMC3003688; doi:10.1371/journal.pone.0015600)
Supplement: Table S2 — Primer sequences used in real-time quantitative PCR. (DOC) [file pone.0015600.s007.doc]

**Supplementary Table 2. Primer sequences**

| **Target Gene** | **Sense primer (5’-3’)** | **Anti-sense primer (5’-3’)** | **Product size (bp)** |
| --- | --- | --- | --- |
| Microtubule Associated Protein 2 (MAP2) | AATAGACCTAAGCCATGTGACATCC | AGAACCAACTTTAGCTTGGGCC | 133 |
| α4 nicotinic acetylcholine receptor (nAChR) | TGGGTACGCAGGGTCTTCC | GCTCAGCCGGCACATCCA | 225 |
| α7 nicotinic acetylcholine receptor (nAChR) | CGCCCATTCCACCTAC | ACCTTTCACTCCTCTTGCC | 257 |
| ISL LIM homeobox 1 (Isl1) | TGTAATCAGTATTTGGACGAG | ATCCCGTACAACCTGATATAATC | 89 |
| Thyrosin Hydroxylase (TH) | ACACCGCCGAGGAGATTG | AGCTGGGGGATATTGTCTTC | 145 |
| M3 muscarinic acetylcholine receptor (mAChR) | Gtctggcttgggtcatctcct | actgctgctgtggtct | 434 |
| Hairy and Enhancer of Split (HES 1) | CGGACATTCTGGAAATGACA | CATTATTGACAGCAGCTGCC | 222 |
| Notch 2 | ACATCATCACAGACTTGGTC | CATTATTGACAGCAGCTGCC | 399 |
| Gamma aminobutyricacid B receptor (GABA B) | GAGATGTTGCTGCTGCTGCTACT | TCAAATAAGACTTGGAGCAGATT | 319 |
| Human PPIA (cyclophilin A) | TGCTGGACCCAACACAAATG | TGCCATCCAACCACTCAGTCT | 80 |
| Vesicular Acetylcholine Transporter (VaChT) | TACGGAGAGCGAAGACGTGAA | ATCATGGCTATGCCAGACGTG | 167 |
| Buturyl Choline Esterase (BuChE) | AAAGCCACAGTCTCTGACCAA | CCCACTGAAGAGCCAACTGT | 401 |
